# Cytocompatibility of boron nanoparticle-modified maxillofacial silicone elastomers: an in vitro study

**DOI:** 10.1186/s12903-026-09254-x

**Published:** 2026-07-15

**Authors:** Esra Nur Avukat, Naim Berker Altuntaş, Canan Akay, Mirac Berke Topcu Ersöz, Handan Sevim Akan, Emre Mumcu

**Affiliations:** 1 Department of Prosthodontics, Faculty of Dentistry, Osmangazi University, Eskişehir, Türkiye; 2 Private Clinic, Eskişehir, Türkiye; 3 Translational Medicine Research and Clinical Center, Osmangazi University, Eskişehir, Türkiye; 4 Advanced Material Technologies Application and Research Center, Osmangazi University, Eskişehir, Türkiye; 5 Department of Biology, Faculty of Science, Hacettepe University, Ankara, Türkiye

**Keywords:** Boron nanoparticles, Cytocompatibility, Maxillofacial silicone, Fibroblasts, Scanning electron microscopy

## Abstract

**Background:**

Boron nanoparticles (BNPs) have attracted increasing interest as additives in dental biomaterials due to their antimicrobial activity, chemical stability, and reinforcing potential. Although various nanoparticles have been incorporated into maxillofacial silicone elastomers to improve mechanical and antimicrobial performance, the cytocompatibility of BNP-modified systems remains insufficiently investigated. This study evaluated the concentration-dependent effects of BNP incorporation on the cytocompatibility of two clinically used maxillofacial silicone elastomers.

**Methods:**

Sixty disc-shaped specimens (2 mm × Ø 5 mm; *n* = 10/group) were fabricated from two room-temperature vulcanizing silicone elastomers (A-2000 and A-2006) incorporating BNPs at 0%, 1 wt%, and 3 wt%. Cytocompatibility was assessed using an MTT assay on human dermal fibroblasts (HDF), which were selected as the test model due to their direct and prolonged contact with maxillofacial prostheses. Material eluates were used for cellular exposure at 24 and 48 h of incubation. Surface morphology and nanoparticle distribution were evaluated by scanning electron microscopy (SEM) to correlate surface characteristics with biological responses.

**Results:**

At 24 h, cell viability in 1 wt% BNP groups was comparable to controls (A-2000-1%: 0.39 ± 0.04 vs. A-2000 control: 0.40 ± 0.04; *p* > 0.05), whereas 3 wt% BNP groups — particularly A-2006-3% (0.21 ± 0.02) — showed significantly reduced viability compared to controls (*p* < 0.001). At 48 h, no statistically significant differences were observed among any group (*p* = 0.26), and all groups exhibited cell viability above the ISO 10993-5 cytotoxicity threshold. A significant within-group decrease in viability from 24 to 48 h was observed in the A-2000 control and A-2000-1% groups (*p* < 0.001), attributable to cell-culture dynamics rather than material cytotoxicity. SEM revealed smooth surfaces in control groups, mild irregularities with dispersed nanoparticle clusters at 1 wt%, and pronounced surface roughness with particulate accumulations at 3 wt%, indicating concentration-dependent nanoparticle agglomeration.

**Conclusions:**

BNP incorporation influenced cytocompatibility in a concentration-dependent manner. Incorporation at 1 wt% preserved cytocompatibility in both silicone systems, whereas 3 wt% was associated with a transient reduction in cell viability, likely related to nanoparticle agglomeration and early burst-release behavior. Differences observed between A-2000 and A-2006 suggest that polymer matrix characteristics modulate the biological effects of nanoparticle incorporation. These findings indicate that BNP concentration and matrix properties are key determinants of early cellular responses in nanoparticle-modified maxillofacial silicone systems.

## Background

Maxillofacial prostheses play a critical role in the rehabilitation of facial defects resulting from trauma, congenital anomalies, or oncological surgeries [1]. The materials used in these prostheses must exhibit adequate mechanical strength, color stability, and biocompatibility to ensure long-term clinical performance [2]. Among available materials, maxillofacial silicone elastomers are widely preferred due to their favorable handling properties, durability, and ability to mimic soft tissue appearance [3]. However, despite these advantages, their clinical longevity remains limited by material degradation, insufficient tear strength, marginal instability, and susceptibility to microbial colonization, particularly by *Candida albicans* [1, 4, 5]. These limitations have led to increasing efforts to modify silicone elastomers to improve both their functional and biological performance.

In recent years, the incorporation of nanoparticles has emerged as a promising approach to enhance the properties of polymer-based maxillofacial materials. Various nanoparticles, including TiO₂, ZnO, and SiO₂, have been shown to improve mechanical strength, surface characteristics, and antimicrobial activity [1, 6–9]. However, despite these benefits, the biological safety of nanoparticle-modified materials remains a major concern. Given that maxillofacial prostheses are in prolonged contact with skin and peri-oral tissues, factors such as nanoparticle size, concentration, composition, and surface chemistry may significantly influence cytotoxic responses [10, 11]. The addition of nanoparticles may alter the polymer network, influence degradation behavior, and potentially increase the release of residual components, thereby affecting cytocompatibility. Therefore, evaluating the cytocompatibility of nanoparticle-modified silicone elastomers is essential prior to their clinical application. In this context, boron nanoparticles (BNPs) are of particular interest, as they may enhance mechanical and antimicrobial properties while potentially influencing biological responses due to their surface activity and interaction with the polymer matrix.

From a broader biomaterials perspective, the release of potentially bioactive or toxic components represents a common underlying concern in polymer-based dental materials [12]. Although substantial research has focused on mechanical performance, biological evaluations—particularly cytotoxicity assessments—remain relatively limited in comparison. This imbalance highlights the need for more comprehensive investigation of the biological behavior of newly developed and modified biomaterials, including nanoparticle-enhanced systems.

Boron-based nanomaterials have recently attracted attention as potential additives in dental and biomedical polymers due to their antimicrobial properties, chemical stability, and reinforcing potential [13–15]. Boron derivatives such as boric acid, boron nitride, and boron nitride nanotubes have been reported to enhance mechanical properties, including flexural strength and hardness, while also contributing to improved surface integrity [13–17]. In addition, certain boron compounds have demonstrated antibacterial effects against oral pathogens [15, 18]. Their low density and high surface area make BNPs attractive for lightweight yet mechanically improved prosthetic materials [18, 19]. Despite these promising characteristics, higher concentrations of boron-based additives may lead to surface alterations, particle agglomeration, or changes in material properties, which could influence biological responses [19, 20].

Despite growing interest in boron-based nanomaterials, their biological behavior within maxillofacial silicone elastomers remains largely unexplored. While various nanoparticles have been incorporated into silicone materials to improve mechanical and antimicrobial performance, evidence regarding the cytocompatibility of BNP-modified systems is still limited. This gap is clinically relevant, as maxillofacial prostheses remain in direct and prolonged contact with soft tissues, where even subtle or transient cytotoxic effects may influence tissue compatibility and patient outcomes [13, 20]. BNPs were selected due to their reported antibacterial activity, chemical stability, and favorable biocompatibility in various dental materials. Although several nanoparticles have previously been incorporated into maxillofacial silicone elastomers, to the authors’ knowledge the biological effects of BNPs within silicone matrices remain largely unexplored [1, 6–9, 18, 21–25]. Therefore, the aim of this study was to evaluate the cytocompatibility of maxillofacial silicone elastomers modified with BNPs at two concentrations (1 wt% and 3 wt%) using the MTT assay at 24 and 48 h. The null hypothesis was that BNP incorporation would not adversely affect fibroblast viability at either time point.

## Methods

### Specimen preparation

**Fig. 1:**
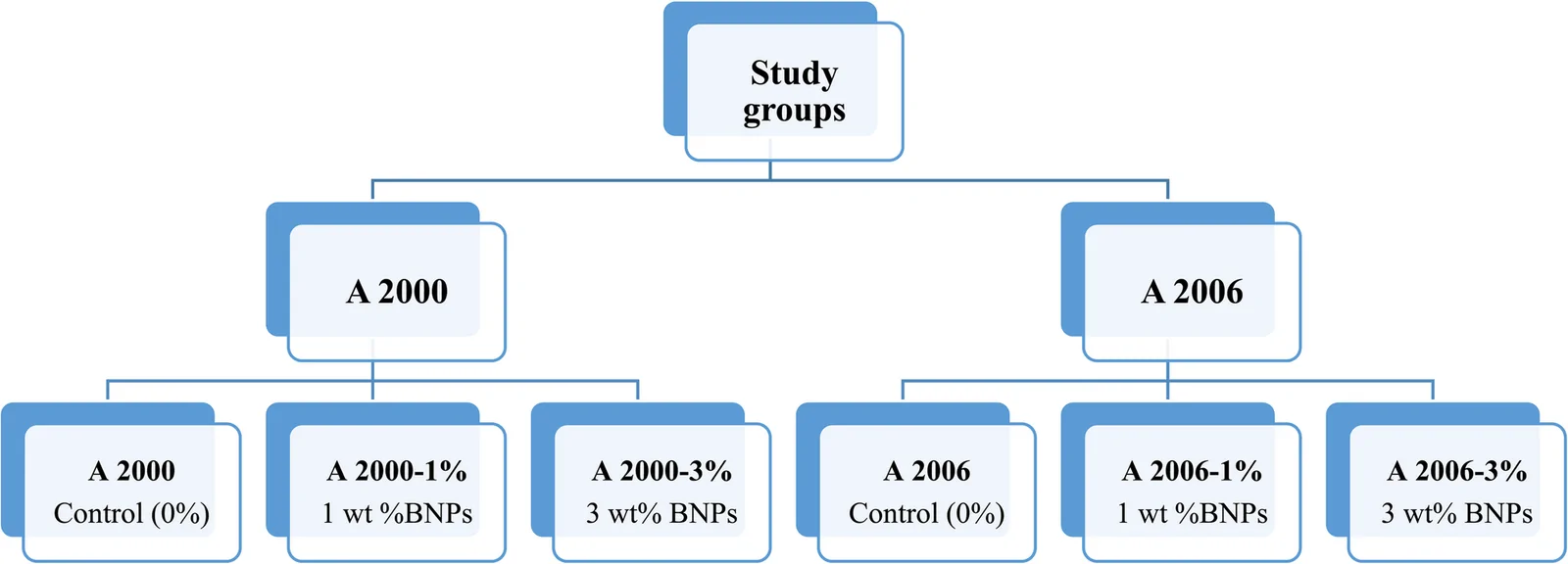
Study design and experimental groups. Six groups were established by combining two room-temperature vulcanizing silicone elastomers (A-2000 and A-2006) with three boron nanoparticle (BNP) concentrations: control (0%), 1 wt%, and 3 wt%. Each group consisted of 10 disc-shaped specimens (*n* = 10 per group; total *N* = 60)

The materials used in the study are presented in Table 1. Two types of room-temperature vulcanizing (RTV) silicone elastomers (A-2000 and A-2006; Factor II Inc., Lakeside, AZ, USA) were used, with parts A and B mixed in a 1:1 ratio according to the manufacturer’s instructions to achieve a homogeneous mixture. The specimen fabrication protocol was adapted from our previous study investigating the mechanical properties of the same silicone elastomers modified with BNPs [20]. A total of 60 disc-shaped specimens (*n* = 10/group) were prepared for MTT testing, incorporating BNPs (BNPs; average particle size: 450 nm; Nanografi Nano Technology, Ankara, Türkiye) at two different concentrations: control (0%), 1 wt%, and 3 wt%. The concentrations were selected based on previous studies that incorporated various nanoparticles into maxillofacial silicone elastomers, most of which used similar concentration ranges [6–9, 13, 18, 20]. For the groups containing BNPs, the nanoparticles were carefully measured using a precision balance before being incorporated into the silicone mixture. This mixture was processed in a vacuum mixer for 2 min and then poured into metal molds while using a vibrator to ensure uniform distribution of the material. Disc-shaped metal molds were used to fabricate specimens with a height of 2 mm and a diameter of 5 mm (2 mm × Ø 5 mm). Following this, the specimens underwent polymerization in a laboratory oven (AS CD-105; Nüve, Ankara, Türkiye) at 60 °C for 6 min. All specimens were fabricated by a single operator (N.B.A.) to maintain consistency in the manual fabrication process. The study groups are shown in Fig. 1.

**Table 1:** Materials used in the study (RTV: room-temperature vulcanizing). All materials were used as received without further modification

Material	Type	Manufacturer	LOT number
RTV silicone elastomer	A-2000	Factor II Inc., Lakeside, AZ, USA	F906842LB
RTV silicone elastomer	A-2006	Factor II Inc., Lakeside, AZ, USA	FL0831212LB
Boron nanoparticles	Boron (450 nm)	Nanografi Nano Technology, Ankara, Türkiye	NG04EO0502

### Cytotoxicity assessment (*MTT assay*)

Cell viability was assessed using the tetrazolium salt 3-[4,5-dimethylthiazol-2-yl]-2,5-diphenyl tetrazolium bromide (MTT; Sigma-Aldrich, Darmstadt, Germany) assay, as described in previous studies [9, 10, 26, 27]. Human dermal fibroblasts (HDF; CC-2511; Lonza, Basel, Switzerland) were cultured in Dulbecco’s Modified Eagle Medium (DMEM) High Glucose supplemented with 2 mM L-glutamine, 10% fetal bovine serum (FBS), and 100 IU/ml penicillin-streptomycin (Biochrom, Berlin, Germany). The test materials were exposed to ultraviolet (UV) light for 30 min prior to the assay to prevent bacterial contamination. All materials were incubated in cell culture media for 24 h at 37 °C, and the resulting eluates were used for the MTT assay. The initial HDF cell density was 1 × 10⁴ cells per well, and the cells were incubated at 37 °C under 5% CO₂. After 24 h of incubation, the medium was replaced with eluates from the test materials, while untreated cells served as controls. The MTT assay was performed at 24 and 48 h of incubation. Briefly, a culture medium containing MTT (Sigma-Aldrich, M5655; Sigma-Aldrich, Darmstadt, Germany) was added to each well and incubated for 4 h. Subsequently, 100 µL of acidic isopropanol (0.05 N HCl in absolute isopropanol) was added to each well at the end of incubation. Absorbance (optical density) was measured at 570 nm using a microplate reader (EZ Read 400; Biochrom, Cambridge, UK). Cell morphology was visualized using an inverted microscope (IX70; Olympus, Tokyo, Japan) equipped with a digital camera (DP71; Olympus, Tokyo, Japan).

### Surface characterization

Surface morphology and nanoparticle dispersion were examined using scanning electron microscopy (SEM). Since silicone specimens are electrically insulating, their surfaces were sputter-coated with a 4 nm layer of gold/palladium using a coating device (EM ACE600; Leica Microsystems, Wetzlar, Germany) to ensure adequate conductivity. The coated specimens were then imaged under a field-emission SEM (Regulus 8230; Hitachi High-Tech Corporation, Tokyo, Japan) at ×1000 magnification to evaluate surface characteristics and BNPs distribution. Images were acquired at an accelerating voltage of 5.0 kV.

### Statistical analysis

The sample size for each experimental group was determined using power analysis software (G*Power, version 3.1.9.7; Heinrich Heine Universität, Düsseldorf, Germany). Assuming a large effect size (f = 0.40), α = 0.05, and β = 0.9, the minimum required sample size per group was calculated as 10. This effect size estimate was based on data from similar studies investigating the cytotoxicity of NP-modified dental materials, which typically report moderate-to-large differences in cell viability [9]. According to Cohen’s conventions, f = 0.40 corresponds to an upper-medium effect size, making it a reasonable assumption for detecting biologically meaningful differences. Statistical analysis was performed using IBM SPSS Statistics (Version 25.0; IBM Corp., Armonk, NY, USA). Normality of data was confirmed using the Shapiro-Wilk test. Within-group differences between 24 and 48 h were analyzed using paired-samples t-tests. One-way ANOVA followed by post hoc Tukey’s test was used for multiple comparisons of cell viability between groups. Statistical significance was set at *p* < 0.001. Artificial intelligence-based language tools (ChatGPT; OpenAI, San Francisco, CA, USA) were used solely for language editing and enhancement of the manuscript. The scientific content is entirely the work of the authors.

## Results

### MTT findings

Table 2 presents the descriptive statistics of the study groups at each time interval. At 24 h, cell viability in the A-2000-1% group (0.39 ± 0.04) was comparable to that of the A-2000 control group (0.40 ± 0.04), with no statistically significant difference (*p* > 0.05). In contrast, the A-2000-3% group (0.31 ± 0.05) exhibited significantly lower viability than the A-2000 control group (*p* < 0.001). Similarly, no significant difference was observed between the A-2006 control and A-2006-1% groups (0.26 ± 0.06) (*p* > 0.05). Among all groups, the A-2006-3% group demonstrated the lowest cell viability at 24 h (0.21 ± 0.02). Cell viability in the A-2000 and A-2000-1% groups was significantly higher than in the remaining groups (*p* < 0.001). No statistically significant difference was observed between the A-2000-3% group and the A-2006 control group (*p* > 0.05).

**Table 2:** Cell viability (mean optical density ± SD) of study groups at 24 and 48 h

Time intervals	A-2000	A-2000-1%	A-2000-3%	A-2006	A-2006-1%	A-2006-3%	t	*p*
24 h	0.4 ± 0.04^a^	0.39 ± 0.04^a^	0.31 ± 0.05^b^	0.32 ± 0.07^ba^	0.26 ± 0.06^bc^	0.21 ± 0.02^c^	57.30	< 0.001*
48 h	0.25 ± 0.03	0.29 ± 0.03	0.30 ± 0.06	0.29 ± 0.07	0.28 ± 0.05	0.26 ± 0.07	1.35	0.26**

Different superscript letters indicate statistically significant differences between groups. *Welch test (24 h); **one-way ANOVA (48 h) with Tamhane’s T2 post hoc test. *t* Test statistic

At 48 h, no statistically significant differences were observed among any of the groups (*p* = 0.26), and all groups showed cell viability values above the ISO 10993-5 cytotoxicity threshold.

Within-group comparisons revealed a significant decrease in cell viability from 24 to 48 h in the A-2000 control (*p* < 0.001) and A-2000-1% groups (*p* < 0.001). No significant changes were observed in the A-2000-3%, A-2006, A-2006-1%, and A-2006-3% groups between the two time intervals (*p* > 0.05), as shown in Table 3.

**Table 3:** Within-group comparisons of cell viability between 24 and 48 h. Paired-samples t- test was used for within-group comparisons. r: correlation coefficient

	24 h (mean ± SD)	48 h (mean ± SD)	*r*	Mean difference ± SD	t-value	*p* ^*^
A-2000	0.4 ± 0.04	0.25 ± 0.03	0.225	0.15 ± 0.05	9.684	**< 0.001**
A-2000-1%	0.39 ± 0.04	0.29 ± 0.03	-0.122	0.1 ± 0.05	6.058	**< 0.001**
A-2000-3%	0.31 ± 0.05	0.3 ± 0.06	-0.611	0.02 ± 0.1	0.540	0.602
A-2006	0.32 ± 0.07	0.29 ± 0.07	-0.156	0.03 ± 0.11	0.900	0.391
A-2006-1%	0.26 ± 0.06	0.28 ± 0.05	-0.315	-0.02 ± 0.09	-0.689	0.508
A-2006-3%	0.21 ± 0.02	0.26 ± 0.07	0.129	-0.04 ± 0.07	-2.042	0.072

### SEM findings

**Fig. 2:**
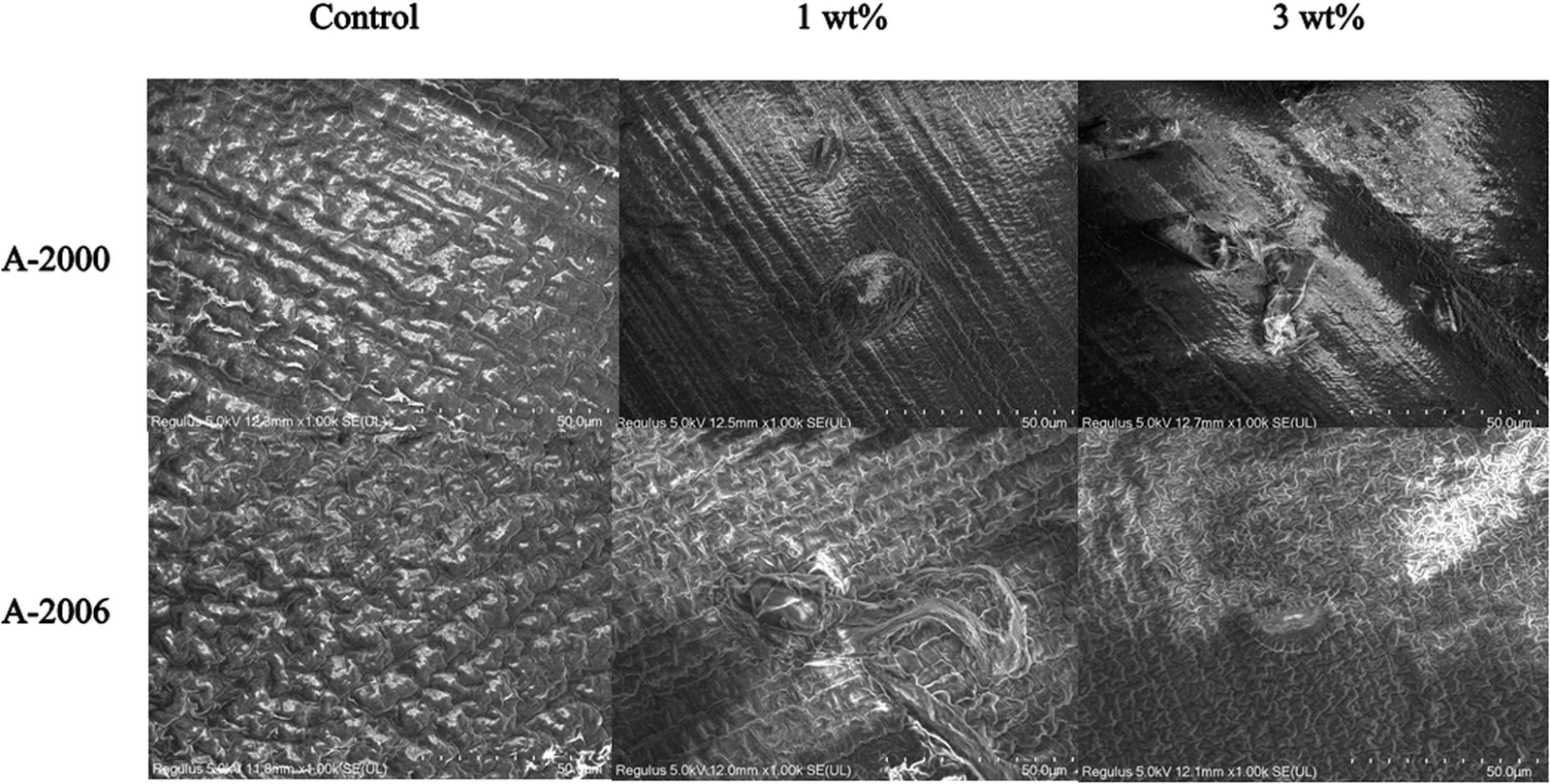
Representative scanning electron microscopy (SEM) images of A-2000 and A-2006 silicone elastomers in control, 1 wt%, and 3 wt% BNP groups. Control specimens exhibited smooth and homogeneous surfaces. The 1 wt% BNP groups showed mild surface irregularities with dispersed nanoparticle clusters, whereas the 3 wt% BNP groups demonstrated increased surface roughness and more pronounced particulate accumulations, indicating greater nanoparticle aggregation

SEM analysis revealed concentration-dependent changes in surface morphology and nanoparticle distribution, as shown in Fig. 2. The control groups of both A-2000 and A-2006 exhibited smooth and relatively uniform surfaces, characteristic of the unmodified silicone matrix. In the 1 wt% BNP groups, mild surface irregularities and small, dispersed nanoparticle clusters were observed. At 3 wt%, the surfaces demonstrated increased roughness and more pronounced particulate accumulations, indicating greater nanoparticle aggregation at higher concentrations. Compared with A-2006, the A-2000 elastomer exhibited fewer surface irregularities across all BNP concentrations.

## Discussion

The null hypothesis of this study was partially rejected. While 1 wt% BNP groups maintained cell viability comparable to controls at both 24 and 48 h, 3 wt% BNP groups demonstrated a statistically significant reduction in viability at 24 h, particularly in the A-2006-3% group (0.21 ± 0.02; *p* < 0.001). However, this reduction was transient, as no significant differences were observed among any group at 48 h (*p* = 0.26). To the authors’ knowledge, this study contributes to the limited evidence on the cytocompatibility of BNP-modified maxillofacial silicone elastomers.

According to ISO 10993-5, cell viability above 70% is considered indicative of non-cytotoxic behavior. In the present study, both A-2000 and A-2006 elastomers containing 1 wt% BNP maintained cell viability above this threshold at both time points, demonstrating acceptable cytocompatibility. At 3 wt%, a transient reduction was observed at 24 h, most notably in the A-2006-3% group, which fell slightly below the non-cytotoxic threshold. By 48 h, all groups recovered to levels above this threshold, suggesting that the observed reduction was mild and not sustained. These findings are consistent with those reported by Tukmachi et al., who found no sustained cytotoxicity in silicone elastomers modified with 1–3 wt% ZrO₂ nanoparticles, further supporting the notion that NP-modified silicone systems can maintain acceptable short-term biocompatibility when concentration is appropriately controlled [18].

The early reduction in cell viability observed in the 3 wt% groups may be explained by an initial release behavior commonly described in NP-containing biomaterials. Pokrowiecki reported that maxillofacial biomaterials often exhibit an initial rapid release of surface-associated particles upon contact with biological fluids, temporarily increasing local concentrations before transitioning to a lower, sustained-release phase [28]. The biphasic response identified in the present study — reduced viability at 24 h followed by full recovery at 48 h — aligns with this behavior, particularly given the pronounced nanoparticle agglomeration zones observed on the silicone surfaces at 3 wt%. Previous studies have similarly reported that nanoparticle clustering and surface heterogeneity can transiently influence material–cell interactions during early exposure periods [14, 29].

Differences in the biological responses between A-2000 and A-2006 silicones may be attributable to intrinsic variations in polymer network structure. Crosslinking density, filler content, and polymer–particle affinity influence nanoparticle distribution, surface availability, and elution profiles. The A-2006 matrix may have facilitated greater early availability of surface-bound BNP clusters, contributing to the more pronounced reduction in viability observed in the A-2006-3% group at 24 h. Nevertheless, the absence of sustained cytotoxicity at 48 h across all groups indicates that both silicone types are biologically acceptable for short-term exposure. Notably, comparable cell viability was observed between the A-2000-3% and A-2006 control groups at 24 h (*p* > 0.05), suggesting that baseline material properties modulate the apparent cytotoxic effects of nanoparticle incorporation.

SEM findings provided morphological support for the biological results. Control specimens exhibited smooth, homogeneous surfaces, while BNP incorporation produced concentration-dependent surface alterations. At 1 wt%, only mild surface irregularities and dispersed nanoparticle clusters were observed, consistent with the absence of cytotoxicity at this concentration. At 3 wt%, pronounced surface roughness and particle aggregation were evident, which may have contributed to the transient reduction in cell viability through increased initial particle exposure. These observations closely parallel the AFM findings from our previous study, which demonstrated increased surface roughness and multimodal height distributions with higher BNP loading [20], confirming that BNP incorporation alters silicone surfaces at both micro- and nanoscales.

The concentration-dependent behavior observed in this study is consistent with previous reports on boron-containing dental materials. Demirci et al. reported that boron-containing composites could enhance the odontogenic and osteogenic differentiation of dental pulp stem cells at low concentrations, while higher concentrations were associated with dose-dependent cytotoxic effects, indicating that cellular responses depend heavily on both concentration and cell type [14]. Similarly, studies on boric-acid-reinforced 3D-printed resins have shown that higher additive concentrations promote microvoid formation, particle clustering, and surface heterogeneity [13]. Collectively, these findings suggest that boron-based additives have an optimal concentration range beyond which particle–matrix interactions may become detrimental, both biologically and mechanically.

The MTT assay was selected for cytotoxicity assessment due to its practicality, reliability, and widespread use in dental biomaterials research [10, 18, 27, 29]. Human dermal fibroblasts were chosen as the test model because dermal tissues are in direct and prolonged contact with maxillofacial prostheses, making them a clinically relevant cell type for this evaluation. From a clinical perspective, the concentration-dependent cytocompatibility profile of BNP-modified silicones is relevant, as excessive nanoparticle loading may not only compromise biological safety but also adversely affect mechanical integrity and color stability — properties critical for long-term prosthetic performance [13, 19, 20]. These considerations highlight the importance of identifying an optimal BNP concentration that balances enhanced material properties with biological safety.

This study has several strengths. It represents one of the first studies to evaluate the cytocompatibility of BNP-modified maxillofacial silicone elastomers, addressing a clear gap in the literature. The use of two silicone systems allowed for comparative analysis of material-dependent biological responses. Standardized specimen fabrication by a single operator and power analysis-based sample size determination further support the methodological rigor of the study.

Nevertheless, several limitations should be acknowledged. First, the incubation periods were limited to 24 and 48 h, which are standard for initial cytotoxicity screening according to ISO 10993-5 but do not capture potential long-term biological responses or delayed effects arising from sustained nanoparticle release. The short observation period was selected to provide foundational data on acute cytotoxicity; however, future studies incorporating extended incubation periods — such as 7 days or longer — as well as dynamic release assays are needed for a more comprehensive evaluation. Second, cytotoxicity was assessed using a single assay; additional assays evaluating oxidative stress, apoptosis, or inflammatory markers would provide a more complete picture of cellular responses. Third, nanoparticle release dynamics were not directly quantified, and such measurements should be incorporated in future studies to better characterize the elution profile of BNP-modified silicone systems.

## Conclusion

Within the limitations of this study, the findings indicate that BNPs incorporation influences the cytocompatibility of maxillofacial silicone elastomers in a concentration-dependent manner. The results further suggest that early cellular responses to nanoparticle-modified silicone materials are governed not only by nanoparticle concentration but also by the characteristics of the polymer matrix.

These findings highlight the importance of considering material–particle interactions when developing nanoparticle-enhanced silicone systems intended for prolonged soft tissue contact. Further studies are required to evaluate long-term biological responses and to better understand the relationship between nanoparticle incorporation and overall material performance.

## Data Availability

The datasets used and analyzed during the current study are available from the corresponding author upon reasonable request. For data access, please contact Dr. Esra Nur Avukat at enavukat@ogu.edu.tr.

## Abbreviations

BNPs: Boron nanoparticles
HDF: Human dermal fibroblasts
MTT: Tetrazolium salt 3-[4,5-dimethylthiazol-2-yl]-2,5-diphenyl tetrazolium bromide
RTV: Room Temperature Vulcanization
SEM: Scanning electron microscopy
UV: Ultraviolet
